# Investigations on the Endemic Species *Taraxacum mirabile* Wagenitz: HPLC–MS and GC–MS Studies, Evaluation of Antioxidant, Anti-Inflammatory, and Antimicrobial Properties, and Isolation of Several Phenolic Compounds

**DOI:** 10.3390/plants13233304

**Published:** 2024-11-25

**Authors:** Seçil Karahüseyin, Nurten Özsoy, Berna Özbek Çelik, Aynur Sarı

**Affiliations:** 1 Department of Pharmacognosy, Faculty of Pharmacy, Cukurova University, 01330 Adana, Türkiye; 2 Department of Pharmacognosy, Institute of Health Sciences, Istanbul University, 34116 Istanbul, Türkiye; 3 Department of Biochemistry, Faculty of Pharmacy, Istanbul University, 34116 Istanbul, Türkiye; nozsoy@istanbul.edu.tr; 4 Department of Pharmaceutical Microbiology, Faculty of Pharmacy, Istanbul University, 34116 Istanbul, Türkiye; berna.ozbek@istanbul.edu.tr; 5 Department of Pharmacognosy, Faculty of Pharmacy, Istanbul University, 34116 Istanbul, Türkiye; aynur@istanbul.edu.tr

**Keywords:** *Taraxacum mirabile* Wagenitz, isolation, HPLC–MS, GC–MS, antioxidant, anti-inflammatory, antimicrobial

## Abstract

In this study, the aim was to investigate the chemical content and in vitro antioxidant, antimicrobial, and anti-inflammatory activities of petroleum ether (PE), dichloromethane (DCM), ethyl acetate (EA), and n-butanol (n-BuOH) fractions obtained from ethanol extracts of the aerial parts and roots of the endemic *Taraxacum mirabile* Wagenitz. This plant is found in the Aksaray–Eskil region and has not been studied in phytochemical studies before. In this context, the chemical content of the aerial parts and root PE fractions was analyzed by GC–MS analysis in terms of terpenes and steroid substances. The composition of phenolic compounds in the aerial parts and root DCM and EA fractions was determined by HPLC–MS analysis. Apigenin, luteolin, and caffeic acid were isolated from the EA fraction of aerial parts. The total amounts of phenolic substances and the DPPH, ABTS, and FRAP antioxidant activities of PE, DCM, EA, and n-BuOH fractions were investigated, and it was found that the fractions had the ability to scavenge DPPH^•^ and ABTS^•+^ radicals, as well as to reduce Fe (III) to Fe (II); however, all of the fractions were significantly less effective (*p* < 0.05) than the reference antioxidant quercetin. Considering that antioxidants can also exert an anti-inflammatory effect, these fractions were evaluated for their ability to inhibit cyclooxygenases (COX-1 and COX-2), the key enzymes of arachidonic acid metabolism that lead to the production of important mediators of inflammation. It was observed that fractions had the ability to inhibit both enzymes, suggesting their possible beneficial effects against inflammation. However, no extract had greater inhibitory activity than the positive control, indomethacin. The antimicrobial activity was determined against different bacterial and fungal strains. It was observed that the aerial parts and root n-BuOH and EA fractions showed weak antibacterial effects. No antifungal activity has been detected against *Candida* sp.

## 1. Introduction

Plants of the genus *Taraxacum* are part of the subfamily Cichorioideae and the tribe Lactuceae under the family Asteraceae, predominantly located in the temperate zones of the northern hemisphere [1,2,3]. In 1920, Stork analyzed this genus and identified approximately 2500 species within it. Since then, numerous new *Taraxacum* species have been identified, resulting in the establishment of over 60 sections, with a total of around 2800 species [3].

**Figure 1:**
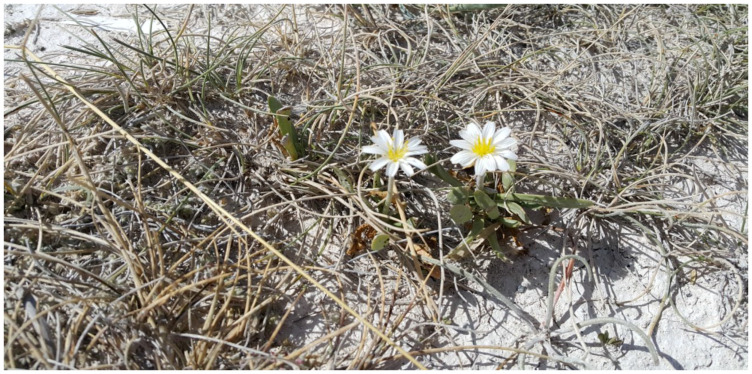
*Taraxacum mirabile* Wagenitz (Photo by: Seçil Karahüseyin).

The species *Taraxacum mirabile* Wagenitz belongs to the Orientalia Hand.-Mazz. section. It is endemic to Central Anatolia in Türkiye. The plant is 3–5 cm tall, and is densely fringed at the base. The leaves are fleshy, glabrous, thin, and narrowly spatulate, featuring a triangular apex. They are either toothless or have small teeth near the apex. The leafless flower stalk is pubescent. The involucre is cylindrical in shape and features short, depressed outer phyllaries. The flowers are not numerous and are white. The achenes are 5–5.5 mm in length, pale brown, not wrinkled, have no beak, and the papus is 5–6 mm (Figure 1). It grows at 800–1300 m [4]. This genus consists of hardy, milk-bearing perennial small plants, represented by 49 species and 54 taxa in Türkiye, which naturally thrive in our country [5,6,7,8]. In Turkish medicine, the plant is utilized as a laxative, diuretic, and potent antidiabetic agent [6]. The plant is recognized for its laxative, cholagogue, and diuretic properties, as well as its efficacy in reducing renal calculi and its antipyretic effects [5,6,9]. Previous phytochemical investigations on *Taraxacum* species yielded a variety of chemicals, including triterpenes, sesquiterpene lactones, indole alkaloids, phytosterols, flavonoids, lignans, phenolic acids, coumarins, beta-carboline alkaloids, and carotenoids [8].

Due to its exceptional biological activity and distinctive biological features, *Taraxacum* sp. is held in the highest respect. Scientists have been able to isolate its active components in recent decades due to its superior pharmacological qualities. Coumarins found in *Taraxacum* sp., such as aesculin, cichoriin, esculetin, scopoletin, etc., have anti-inflammatory, anti-coagulant, bacteriostatic, and anticancer activities; flavonoids found in *Taraxacum* sp., such as quercetin, chrysoeriol, diosmetin, luteolin, etc., typically have antioxidative activity. Sesquiterpene lactones, such as cichorioside C, sonchuside, taraxafolide, ixerin D, and others, are the primary sesquiterpene chemicals found in *Taraxacum* sp. These compounds possess anti-inflammatory and antibacterial properties. *Taraxacum* sp. contains triterpenoids and sterols that can help reduce the symptoms of cardiovascular illnesses. These include α-amyrin acetate, β-amyrin acetate, lupenyl acetate, daucosterol, β-sitosterol, and others [10].

The scientific literature has documented twelve medicinal qualities of *Taraxacum officinale*. These qualities encompass diuretic, hepatoprotective, anticolitic, immunoprotective, antiviral, antifungal, antibacterial, antiarthritic, antidiabetic, antiobesity, antioxidant, and anticancer actions. The medicinal properties of *Taraxacum officinale*, predominantly documented in the scientific literature, include antioxidant, hepatoprotective, and anticancer activity [11]. Mice that were given dandelion extract by mouth had less lung damage caused by LPS, and the amount of TNF-α and IL-6 in their bronchoalveolar lavage fluid went down. The ear swelling caused by 12-O-tetradecanoylphorbol-13-acetate was greatly reduced by dandelion extracts from *T. officinale* and *T. platycarpum*. The oral treatment of *Taraxacum* herb extracts inhibited dextran sulfate sodium-induced colitis in mice by decreasing inflammatory mediators via the inactivation of extracellular signal-regulated kinases and the inhibition of NF-kB, signal transducer, and activator of transcription 3. The water extract of *T. officinale* leaves greatly reduced the levels of TNF-α and ICAM1 in LPS-induced rat mammary microvascular endothelial cells at concentrations of 100–200 μg/mL. Giving the dandelion leaf extract to female BALB/c mice by mouth also improved mastitis caused by *Staphylococcus aureus*. Additionally, in Madin–Darby canine kidney (MDCK) and human lung adenocarcinoma (A549) cells, the dandelion extract stopped the growth of influenza virus type A by preventing the virus from replicating [2].

The main aim of this study is to understand whether the *T. mirabile* species, endemic to our country, has the same compounds as the other *Taraxacum* species that have been examined and whose specific activities have been identified, and whether it has any biological activity. For this purpose, the chemical composition and in vitro antioxidant, antimicrobial, and anti-inflammatory activities of the endemic *T. mirabile* species collected from the Aksaray–Eskil region were investigated, and the results are presented in this study.

## 2. Results

### 2.1. Isolated Phenolic Compounds

The EA20–29 fraction gave us TMH-1 (5 mg), the EA41–56 fraction gave us TMH-2 (5 mg), and the EA148–155 fraction gave us TMH-17 (6 mg) (Supplementary Materials, Table S1 and Figure S1). We obtained these by using a dichloromethane and methanol gradient system for silica gel column chromatography.

### 2.2. Results of HPLC–MS for Ethyl Acetate Fractions of Aerial Parts and Roots

We selected DCM and EA fractions for the HPLC–MS study of polar chemicals (flavonoids, phenolic compounds, coumarins, etc.) due to the elevated concentration of phenolic compounds in polar solvents. The aerial parts and root EA fractions of *T. mirabile* were analyzed for phenolic compounds, and the HPLC–MS results are shown in Table 1.

The substances found in the aerial EA fraction are epigallocatechin, trans-taxifolin, apigenin-7-glucoside, apigenin, caffeic acid, chicoric acid, dihydrokaempferol, fumaric acid, herniarin, hispidulin, luteolin-7-rutinoside, naringenin, rosmarinic acid, verbascoside, luteolin, and luteolin-7-glucoside.

The substances found in the root EA fraction are epigallocatechin, apigenin-7-glucoside, apigenin, caffeic acid, chicoric acid, dihydrokaempferol, fumaric acid, hispidulin, isosakuranetin, luteolin-7-rutinoside, naringenin, rosmarinic acid, verbascoside, luteolin, and luteolin-7-glucoside.

### 2.3. Results of HPLC–MS for Aerial Parts and Root Dichloromethane Fractions

The aerial parts and root DCM fractions of *T. mirabile* were analyzed for phenolic compounds, and HPLC–MS results are shown in Table 2.

The substances found in the dichloromethane fraction of the aerial parts are chlorogenic acid, fumaric acid, verbascoside, chicoric acid, caffeic acid, vanillic acid, salicylic acid, luteolin, apigenin, hispidulin, and chrysin.

The substances found in the root dichloromethane fraction are chlorogenic acid, fumaric acid, verbascoside, chicoric acid, caffeic acid, vanillic acid, salicylic acid, luteolin, apigenin, hispidulin, and chrysin.

### 2.4. Aerial Parts Petroleum Ether Fraction GC–MS Findings

**Figure 2:**
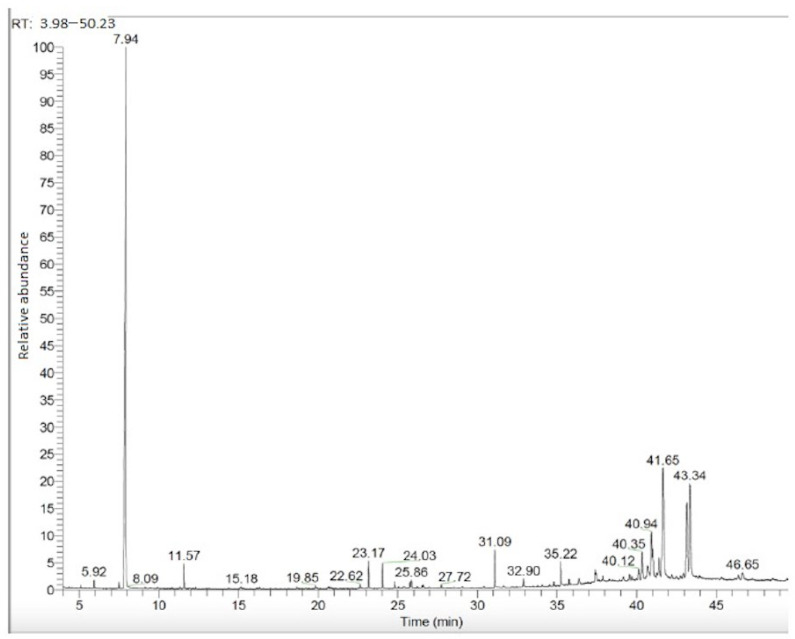
Aerial parts PE fraction GC–MS chromatogram.

The GC–MS analysis of nonpolar compounds (fatty acids, terpene compounds, etc.) favors petroleum ether extracts due to their elevated concentration of terpene compounds in apolar solvents. The GC–MS chromatogram obtained from the aerial parts PE fraction is given in Figure 2. The compounds detected according to the chromatogram results are given in Table 3 together with their retention times and content (%).

### 2.5. Root Petroleum Ether Fraction GC–MS Findings

**Figure 3:**
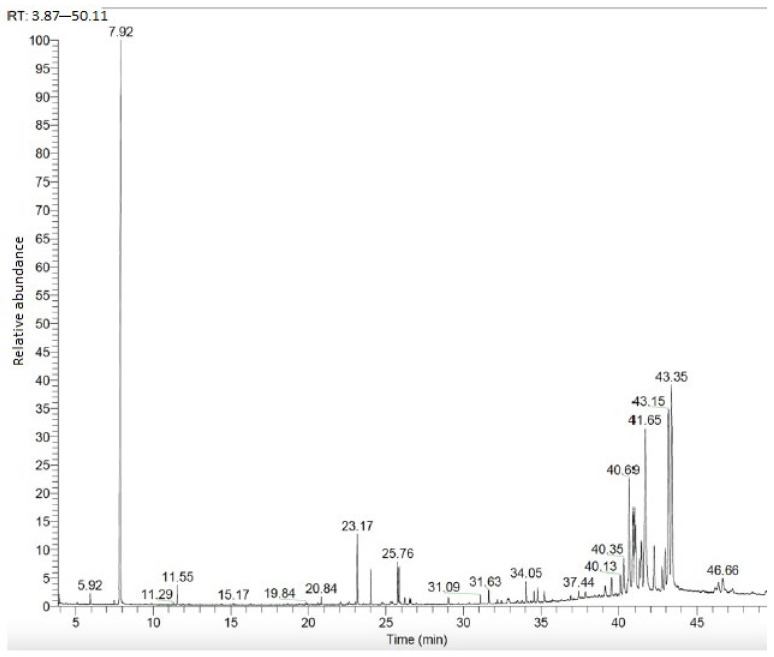
Root PE fraction GC–MS chromatogram.

The GC–MS chromatogram obtained from the root PE fraction is given in Figure 3. The compounds detected according to the chromatogram results are given in Table 4 together with their retention times and content (%).

### 2.6. Anti-Inflammatory Activity

The anti-inflammatory activities of the PE, DCM, EA, and n-BuOH fractions obtained from the aerial parts and roots of the *T. mirabile* plant were evaluated against the key inflammation-related enzymes cyclooxygenase-1 (COX-1) and cyclooxygenase-2 (COX-2). The ability of the fractions to inhibit COX-1 and COX-2 was determined by calculating the percentage inhibition of prostaglandin production measured by the enzyme immunoassay. The results showed significant inhibitory activities of up to 96% against both enzymes at 10 mg/mL, as presented in Table 5, indicating that this species could be a potential source of plant-derived anti-inflammatory substances. However, no extract had greater inhibitory activity than the positive control, indomethacin.

### 2.7. Antimicrobial Activity

According to the results of the antimicrobial activity, it was observed that the aerial EA and aerial BuOH fractions, as well as the root EA and root BuOH fractions, showed a weak effect, albeit to a small extent, on the strains of *Staphylococcus aureus*, *Proteus mirabilis*, and *Enterococcus faecalis*. However, no activity has been observed against *Candida* species.

### 2.8. Antioxidant Activity

The antioxidant potential of the fractions was determined by evaluating the scavenging effect on DPPH and ABTS radicals as well as Trolox equivalent antioxidant capacity (TEAC) and ferric ion reducing antioxidant power (FRAP). Table 6 presents the results of the antioxidant activities, expressed as the EC_50_, TEAC, and FRAP values of the fractions under study.

Based on the EC_50_ values, aerial parts and root EA fractions showed the highest scavenging effect on DPPH and ABTS radicals, followed by the DCM fraction, while the BuOH fraction showed the lowest activity. However, when compared with the EC_50_ values of quercetin, the radical scavenging activities of the above-mentioned four fractions were found to be significantly lower (*p* < 0.05).

The Trolox equivalent antioxidant capacity (TEAC) at 10 mg/mL was highest for the EA and DCM fractions of the aerial parts (*p* > 0.05) and similar to the TEAC value of quercetin at 0.31 mg/mL (*p* > 0.05). On the other hand, the TEAC values of the EA, DCM, and BuOH fractions of the roots were significantly lower (*p* < 0.05).

The FRAP values at 10 mg/mL were the highest and similar for aerial and root EA and aerial part DCM fractions (*p* > 0.05), while the corresponding values for BuOH and PE fractions were lower (*p* < 0.05). The FRAP values of the aerial parts and root EA and aerial parts DCM fractions at 10 mg/mL were comparable to the FRAP value of quercetin at the concentration of 0.31 mg/mL (*p* > 0.05).

None of the fractions significantly outperformed quercetin, despite their ability to scavenge radicals and reduce Fe^3+^.

### 2.9. Results of Total Phenolic Compounds Determination

The examined fractions contained total phenolic compounds ranging from 8.25 to 49.53 mg GAE/g extract. EA and DCM fractions contained the highest quantity of phenolic compounds. BuOH was less effective at extracting phenolic compounds. We found the lowest amount in the aerial parts and root PE fractions.

The variation in the activity of the fractions could be attributed to differences in the proportion of active components responsible for the tested activities, which could result from differences in their solubility in different solvents. Solvents with relatively higher polarity are generally more efficient for extracting phenolic compounds. EA produced the highest yield of crude extract. EA is a moderately polar solvent, and therefore it has the ability to extract both polar and nonpolar compounds. EA is often used as an extraction solvent with significant selectivity to extract low-molecular-weight phenolic compounds and high-molecular-weight polyphenols.

The results indicated that phenolic compounds are present in higher concentrations in the aerial parts than in the roots.

## 3. Discussion

We studied the phytochemicals in the PE, DCM, and EA parts of the ethanol extracts of *T. mirabile*’s aerial parts and roots, which is a native species. Our studies were mostly interested in terpenes (by GC–MS), steroids (by GC–MS), and phenolic compounds (by HPLC–MS and isolation). Simultaneously, we examined the anti-inflammatory, antimicrobial, and antioxidant properties of the PE, DCM, EA, and n-BuOH fractions extracted from the roots and aerial parts using ethanol. We also found out how much total phenolic content each fraction had. This species has undergone a thorough study for the first time in the literature.

We evaluated the PE fractions from the aerial and root ethanol extracts of the *T. mirabile* species for their content of fatty acids, sterols, and triterpenic compounds using GC–MS analysis. The GC–MS test found 12 different substances in the aerial PE fraction. Of these, two were fatty acids (palmitic acid and linoleic acid), four were sterols (campesterol, β-sitosterol, clionasterol, and lanosterol), and six were triterpenes (α-amirin, lupeol, taraxasterol, betulin, α-amirin acetate, and lupeol acetate). GC–MS analysis found 16 substances in the root PE fraction. Two of them were fatty acids (palmitic acid and linoleic acid), five were steroids (campesterol, stigmasterol, β-sitosterol, clionasterol, and lanosterol), and nine were triterpenes (germanicol, α-amirin, lupeol, taraxasterol, taraxerol, epi-psi-taraxastanonol, betulin, α-amirin acetate, and lupeol acetate). According to these results, the compounds found in the PE fractions derived from the ethanol extracts of the aerial parts and roots align with the data in the literature on *Taraxacum* species previously studied [12,13]. In a 2011 study by Jung et al., GC–MS analysis of apolar extracts from all plant materials of the species *T. officinale*, *T. coreanum*, and *T. platycarpum* identified 27 compounds, including stigmasterol, β-sitosterol, β-amirin, and lanosterol, along with some amino acids and carbohydrates [12]. In another study in 2015, Huber et al. identified β-amirin acetate, cycloartenol acetate, α-amirin acetate, and lupeol acetate compounds in *T. officinale* latex [14]. Another study from 2018 used GC–MS to identify 30 compounds in the n-hexane extract of the aerial parts of *T. officinale*, including β-sitosterol, α-amirin, β-amirin, lupeol acetate, taraxasterol acetate, and cycloartenol acetate [15]. Grauso et al.’s 2019 study used GC–MS to detect fatty acids in the ethanol extract of *T. officinale* leaves, while Sasikala et al.’s 2019 study identified phenols, alkanes, and alkenes in the same ethanol extract [13,16].

HPLC–MS analysis identified the phenolic compounds present in the DCM fractions of the aerial parts and root ethanol extracts of the *T. mirabile* species. HPLC–MS analysis identified 11 compounds in the DCM fraction from the ethanol extract of the aerial parts, including chlorogenic acid, fumaric acid, verbascoside, chicoric acid, caffeic acid, vanillic acid, salicylic acid, luteolin, apigenin, hispidulin, and chrysin. We identified a total of 11 compounds in the DCM fraction from the root ethanol extract, including chlorogenic acid, fumaric acid, verbascoside, chicoric acid, caffeic acid, vanillic acid, salicylic acid, luteolin, apigenin, hispidulin, and chrysin. Accordingly, the compound present in the highest amount in the aerial parts DCM fraction is luteolin, while the compound present in the highest amount in the root DCM fraction is vanillic acid. These results have shown that the DCM fractions of the plant contain phenolic compounds.

We used the EA fractions from the ethanol extracts of the plant’s aerial parts and roots for the isolation and HPLC–MS analysis of phenolic compounds. The HPLC–MS analysis detected 16 compounds in the aerial parts EA fraction, including epigallocatechin, trans-taxifolin, apigenin, apigenin-7-glucoside, caffeic acid, chicoric acid, dihydrokaempferol, fumaric acid, herniarin, hispidulin, luteolin-7-rutinoside, naringenin, rosmarinic acid, verbascoside, luteolin-7-glucoside, and luteolin. We identified a total of 15 compounds in the root EA fraction, which included epigallocatechin, apigenin, apigenin-7-glucoside, caffeic acid, chicoric acid, dihydrokaempferol, fumaric acid, hispidulin, isosakuranetin, luteolin-7-rutinoside, naringenin, rosmarinic acid, verbascoside, luteolin-7-glucoside, and luteolin. Accordingly, the compound present in the highest amount in the aerial parts EA fraction is luteolin-7-rutinoside, while the compound present in the highest amount in the root EA fraction is verbascoside. Based on these results, we found the isosakuranetin compound in the root EA fraction, but not in the aerial parts EA fraction. The root EA fraction contained the isosakuranetin compound, despite the absence of trans-taxifolin and herniarin compounds. These results confirm the richness of phenolic compounds in the EA and DCM fractions obtained from the ethanol extracts of the aerial parts and roots and confirm their compatibility with the data in the literature [17]. Shi et al. identified 32 phenolic compounds in the methanol extract of *T. mongolicum* aerial parts, according to the HPLC–DAD–RSD–ESI–MS results [17]. The HPLC–DAD–RSD–ESI–MS/MS method identified 18 flavonoids and phenolic acids in the study of the fresh plant methanol extract of *Neo-T. siphonanthum* [18]. Another study using *T. officinale* leaf ethanol extract detected polyphenolic compounds [18]. In a different study, HPLC and GC–MS were used to find out what chemicals were in the methanol extract of *T. officinale* root latex. They found inositol esters and sesquiterpene lactone glycosides [14].

Isolation studies on the aerial EA fraction of the plant resulted in the pure isolation of apigenin, luteolin, and caffeic acid compounds. We purified these compounds using TLC and column chromatography and performed UV shift analyses on flavonoid-structured compounds, first obtaining their spectra with a UV spectrophotometer. Consequently, ^1^H-NMR analysis verified the substances. Additionally, the obtained substances are consistent with the findings of the HPLC–MS analysis.

In Turkish traditional medicine, *Taraxacum* is well-known for its laxative, diuretic, and potent antidiabetic properties, and its use in treating eye disease. Many researchers have focused on *Taraxacum*’s therapeutic potential, as outlined in the review by Schütz (2006) and Di Napoli and Zucchetti (2021) [6,11].

Researchers have identified the role of reactive oxygen species (ROS)-mediated oxidative damage in various pathological disorders in recent years. Consequently, augmenting the body’s antioxidant and free radical scavenging capacity is essential for the prevention and management of disorders, including inflammation and cancer. Natural antioxidants have garnered considerable attention as prospective therapeutic agents owing to their capacity to mitigate oxidative stress and inflammation. Previous research has shown that some species of *Taraxacum* have strong antioxidant and free radical scavenging abilities. These properties may help protect against diseases caused by oxidative stress by counteracting their harmful effects [19,20,21,22]. In this study, we found that *T. mirabile* reduces Fe^3+^ and transforms stable synthetic radicals such as DPPH and ABTS into less reactive species by donating electrons or hydrogen, which aligns with the existing literature.

The EA and DCM fractions, extracted from the aerial parts and roots of *T. mirabile*, exhibit the most promising antioxidant and anti-inflammatory properties. Researchers have already found that polyphenolic compounds such as luteolin, luteolin-7-glucoside, caffeic acid, chicoric acid, rutin, and apigenin give the extracts their antioxidant properties [21].

COX inhibitors have been recognized as a useful method for treating inflammation. Non-steroidal anti-inflammatory drugs (NSAIDs) stop the production of inflammatory mediators from arachidonic acid by blocking COX-1 and COX-2 enzymes. This makes them the most popular way to treat inflammation, but long-term use is linked to adverse effects. It is promising to discover alternative medicinal agents that exhibit comparable efficiency with reduced adverse effects. COX-2 inhibitors have demonstrated antitumor effects against several cancer types, making them a target for numerous cancer-preventive medications. In this study, the results showed that fractions can reduce inflammation by blocking COX-1 and COX-2. They may one day be used instead of traditional NSAIDs to treat diseases that are linked to inflammation. The results demonstrated consistency with existing findings regarding the efficacy of *Taraxacum* species as anti-inflammatory agents. Chinese phytomedicine extensively utilizes *T. mongolicum*, which has documented anti-inflammatory properties. This species may facilitate the advancement of novel anti-inflammatory and anticancer pharmaceuticals [23].

In the antimicrobial activity study, it was determined that the aerial parts n-BuOH fraction showed an MIC value of 1250 mg/mL against *P. mirabilis*, *E. faecalis*, and *S. aureus* species; the aerial parts EA fraction showed activity against *S. aureus*; the root n-BuOH fraction showed activity against *S. aureus*; and the root EA fraction showed weak antibacterial activity against *E. faecalis* and *S. aureus*. No antifungal activity indicator has been detected against *Candida* species. Researchers have found that *Taraxacum* extracts possess antimicrobial activity against various bacteria, yeasts, and fungal strains. Researchers tested a 40% ethanol extract from *T. officinale* leaves against three bacterial strains: *Staphylococcus aureus*, *Escherichia coli*, and *Salmonella abony enterica*. Both undiluted and diluted extracts showed antimicrobial activity against these microorganisms. The polyphenolic fraction of dandelion leaf extracts demonstrated a higher level of antimicrobial activity, with extracts at a concentration of 1 mg/mL demonstrating moderate inhibitory activity against *B. subtilis* and low inhibitory activity against *E. coli* [15,24].

Traditional medicine typically uses whole plants instead of isolated pure compounds. Generally, the isolated compounds are more active at a very low concentration than the extract at an equivalent dose; to reach a similar extent of activity, the concentration required for the extract may be significantly higher than that required for the pure compound. Although the tested fractions’ activities were significantly lower (*p* < 0.05) than the activities obtained for the reference compound, it was evident that the extracts did show antioxidant, anti-inflammatory, and antimicrobial activities.

## 4. Materials and Methods

### 4.1. Plant Material

The *T. mirabile* plant was collected on 31 July 2016 from Aksaray–Eskil, at the Eskil old garbage-disposal site. The specimen of the plant was identified and recorded at the Istanbul University Faculty of Pharmacy Herbarium with the code ISTE 115021 by Assoc. Prof. Dr. Münevver Bahar Gürdal Abamor, a member of the Department of Pharmaceutical Botany.

### 4.2. Extraction

*T. mirabile* species were desiccated in the shade, with the aerial parts and roots being segregated and ground; thereafter, they were percolated individually with ethanol (EtOH-Merck 1.00983.2500) at ambient temperature, commencing with the aerial parts followed by the root segments. The collected ethanol parts were condensed with the help of a Büchi Rotavapor R-200/Büchi Pump V-700 (Buchi Labortechnik AG, Industriestrasse 9, CH-9230, Flawil, Switzerland) at a temperature not exceeding 50 °C and at low pressure, and the same parts were combined. In this way, ethanol extracts of the aerial parts and then the root parts were obtained. The aerial ethanol and root ethanol extracts were separately dissolved in methanol (Merck 1.06009.2511) and distilled water (1:2) and taken into a separatory funnel, and then fractionated on the basis of polarity increment by consuming petroleum ether (PE) (Merck 1.01775.5000), dichloromethane (DCM) (Merck 1.06050.2500), ethyl acetate (EA) (Merck 1.09623.2500), and n-butanol (BuOH) (Merck 1.01990.2500), respectively.

### 4.3. Chromatography Methods Used in Isolation

This study used silica gel [Merck Silica Gel 60 (0.063–0.200 mm 70–230 mesh)] (Merck KGaA Frankfurter, Str. 250 64293, Darmstadt, Germany) as the adsorbent for column chromatography to separate the obtained fractions. We used sephadex (GE Healthcare Sephadex LH-20) (Sigma-Aldrich, St. Louis, MO, USA) as the adsorbent to purify the substances in the obtained fractions. We used thin-layer chromatography to check fractions obtained from sephadex and silica gel column chromatographies. We used silica gel aluminum plates (Merck 1.05554.0001, TLC Silica gel 60 F_254_ 20 × 20 cm) (Merck KGaA Frankfurter, Str. 250 64293, Darmstadt, Germany) to combine fractions carrying similar substances or to control purified substances with reference substances.

### 4.4. Isolation of Some Phenolic Compounds

Approximately 30 g of the aerial parts EA fraction was subjected to coarse separation by silica gel column chromatography using an appropriately sized glass column. The column chromatography started with dichloromethane and continued with a gradual increase of methanol through a gradient system. NMR analyses were performed by dissolving the substances with deutero methanol (CD_3_OD) in a BRUKER BIOSPIN brand AVANCE III 400 MHz model nuclear magnetic resonance device (Bruker Corporation 19 Hartwell Avenue Billerica, MA, USA) at Giresun University Central Laboratory.

### 4.5. High Pressure Liquid Chromatography–Mass Spectroscopy (HPLC–MS)

HPLC–MS analyses were performed by ESI–MS (electrospray ionization mass spectroscopy) spectroscopy on the Thermo ORBITRAP Q-EXACTIVE brand model HPLC device (Thermo Fisher Scientific 168 Third Avenue, Waltham, MA, USA) at Bezmialem Vakıf University Drug Application and Research Center. In this study, we conducted research on phenolic compounds, introducing 61 standard substances (51 of them are phenolic compounds, 10 of them are compounds from other groups) one by one to the system before preparing the fractions at the required concentrations. We then conducted scans in MS with a mass/charge ratio ranging from 100 to 900. We determined the fractions of the substances matching the standards during this period. All the standards used in the analysis are products of Sigma-Aldrich. The conditions were as follows: mobile phase A: 1% formic acid–water; mobile phase B: 1% formic acid–methanol; column Troyasil C18 HS-150 × 3 mm 5 μ; ion source: ESI; mass scanning range: 100–900 *m*/*z*; spray voltage (kV): 3.80; capillary temperature: 320 °C. This was employed in the gradient method (Table 7) as described below:

### 4.6. Gas Chromatography–Mass Spectroscopy (GC–MS)

GC–MS analyses were performed by gas chromatography–mass spectrometry on a Thermo Finnigan Trace DSQ GC–MS instrument with an EI (electron ionization) detector and auto-injector (AS 3000 Liquid Autosampler, Thermo Fisher Scientific, Milan, Italy) at Istanbul University-Cerrahpaşa Advanced Analyses Laboratory and Wiley Library for identification. The conditions were as follows: column ZB-5MS (5% polysyryarylene-95% polydimethylsiloxane) 30 × 0.25 × 0.25 mm (length × ID × film thickness); carrier gas: helium; column flow rate: 1 mL/min; injection temperature: 300 °C; column temperature: 80 °C for 1 min and 300 °C for 5 min; injection quantity: 2 μL; ion source temperature: 200 °C; mass range: 1–1000 *m*/*z*.

We set the oven temperature to 80 °C initially and maintained it there for 1 min. At the end of this period, we rapidly raised the oven temperature to 300 °C at a rate of 6 °C/min and maintained this temperature for 5 min. Helium served as the carrier gas, maintained at a constant flow rate of 1 mL/min. The mass selective detector operated in electron impact (EI) mode with an electron energy of 70 eV.

### 4.7. Anti-Inflammatory Activity Assay

We used an enzyme immunoassay (EIA) kit (CAYMAN Chemical 560131, Ann Arbor, MI, USA) to perform the cyclooxygenase inhibitory assay. The manufacturer’s protocol was followed.

### 4.8. Antimicrobial Activity Assay

We tested the extracts for antifungal and antibacterial activity using the microdilution method, which is in line with the criteria set by the Clinical Laboratory Standards Institute (CLSI) [25,26]. The microorganisms used during the experiment and their American Type Culture Collection (ATCC) codes are as follows:

Gram-positive standard bacteria: *Staphylococcus aureus* ATCC 29213, *Staphylococcus epidermidis* ATCC 12228, and *Enterococcus faecalis* ATCC 29212.

Gram-negative standard bacteria: *Escherichia coli* ATCC 25922, *Klebsiella pneumoniae* ATCC 4352, *Pseudomonas aeruginosa* ATCC 27853, and *Proteus mirabilis* ATCC 14153.

Yeast-type fungi species: *Candida albicans* ATCC 10231, *Candida parapsilosis* ATCC 22019, and *Candida tropicalis* ATCC 750.

For Gram-positive and Gram-negative bacteria, 5 × 10^5^ colony-forming units (cfu)/mL were prepared from 4–6 h of fresh culture in Mueller–Hinton Broth (Difco, Detroid, MI, USA) medium. For *Candida* species, it was prepared as 0.5–2.5 × 10^3^ cfu/mL from 24 h culture in Sigma-Aldrich RPMI-1640 medium. The inoculated microplates were incubated at 35 °C for 18–24 h for bacteria and at 35 °C for 46–50 h for *Candida* species. The lowest concentration of extract or standard substance without visible growth was determined as the minimum inhibition concentration (MIC) value.

### 4.9. Antioxidant Activity Assay

#### 4.9.1. DPPH Radical Scavenging Activity

The DPPH activity assay was performed based on the method developed by Brand-Williams et al. [27]. Different amounts of quercetin and fractions (0.1 mL) were added to 3.9 mL of a DPPH solution that was 6 × 10^−5^ M in methanol. We incubated the mixture in darkness at ambient temperature for 30 min. We subsequently quantified the reduction in absorbance of the resultant solution spectrophotometrically at 517 nm relative to methanol. We conducted all measurements in triplicate and then averaged them.

#### 4.9.2. Total Antioxidant Capacity Assay

The total radical antioxidant potential of the fractions was measured using the Trolox equivalent antioxidant coefficient (TEAC) assay as described by Re et al. with minor modifications [28]. To generate ABTS*^+^, we maintained a combination of 7 mM ABTS stock solution and 2.45 mM potassium persulfate in the dark at room temperature for 12 to 16 h. We then included the ABTS*^+^ solution into the fraction. At precisely the sixth minute after the initial mixing, we noted a reduction in absorbance at 734 nm.

#### 4.9.3. Ferric-Reducing Antioxidant Power (FRAP) Assay

The FRAP assay was performed using the methodology established by Benzie and Strain [29]. We freshly synthesized the FRAP reagent, which consists of 10 mM TPTZ in 40 mM HCl, 20 mM FeCl_3_, and 0.3 M acetate buffer (pH 3.6; ratio 1:1:10), and incubated it at 37 °C. After adding the FRAP reagent to the fraction, we recorded the absorbance at 593 nm at the fourth minute. The reduction power was determined by the standard curve derived from various concentrations of FeSO_4_·7H_2_O. We presented the findings as mM Fe^2+^ equivalents.

#### 4.9.4. Determination of Total Phenolic Compounds

The total phenolic component concentration in the fractions was assessed using the Folin–Ciocalteu reagent [30]. We incorporated the Folin–Ciocalteu solution into the fractions and allowed them to incubate for 3 min. We then combined a 2% Na_2_CO_3_ solution and incubated it for an additional 2 h. We measured the absorbance of the blue mixture at 760 nm. We expressed the concentration of phenolic components as mg of gallic acid equivalents (GAE) per gram of extract.

#### 4.9.5. Statistical Assessment

The data obtained from the study are presented as mean ± standard deviation. In the evaluation of the difference between the tested items, Student’s *t*-test was utilized. The significance threshold has been set as *p* < 0.05. The relationship between the variables was evaluated using correlation analysis.

## 5. Conclusions

This study has enriched our knowledge of the phytochemical compounds in the aerial and root parts of *T. mirabile*. This study has also identified and reported the presence of fatty acids, sterols, triterpenes, and phenolic compounds in the aerial and root parts of *T. mirabile* for the first time. The study has demonstrated the anti-inflammatory, antimicrobial, and antioxidant activities of the fractions obtained from the aerial and root parts of *T. mirabile*. It was concluded that the extracts have the potential to be beneficial antioxidants and anti-inflammatory agents.

## Figures and Tables

**Table 1:** Phenolic compounds determined by HPLC–MS in EA fractions.

Compound Name	*m*/*z*	Ionization Mode	Aerial Parts (mg/L)	Root (mg/L)	LOD/LOQ (mg/L)	U % (k = 2)
Epigallocatechin	305.0666	Negative	0.46	0.55	0.12/0.40	3.11
Trans-taxifolin	303.0510	Negative	0.11	<LOD	0.14/0.47	2.97
Apigenin-7-glucoside	433.1129	Positive	16.21	0.78	0.18/0.60	3.13
Apigenin	271.0601	Positive	29.41	2.04	0.22/0.72	2.72
Caffeic acid	179.0349	Negative	29.10	69.78	0.19/0.62	2.41
Chichoric acid	473.0725	Negative	36.93	9.75	0.14/0.48	1.53
Dihydrokaempferol	287.0561	Negative	54.39	1.23	0.11/0.36	3.80
Fumaric acid	115.0036	Negative	58.62	10.79	0.26/0.88	3.15
Herniarin	177.0546	Positive	13.62	<LOD	0.17/0.58	0.94
Hispidulin	299.0561	Negative	5.38	0.90	0.14/0.46	1.73
Isosakuranetin	285.0768	Negative	<LOD	3.65	0.23/0.77	1.21
Luteolin-7-rutinoside	593.1511	Negative	623.86	30.13	0.22/0.73	1.43
Naringenin	271.0612	Negative	0.83	0.99	0.2/0.67	4.15
Rosmarinic acid	359.0772	Negative	30.38	10.93	0.2/0.68	4.38
Verbascoside	623.1981	Negative	18.81	221.08	0.46/1.53	3.59
Luteolin-7-glucoside	447.0933	Negative	24.034	0.086	0.01/0.03	11.29
Luteolin	285.0405	Negative	53.96	3.499	0.01/0.03	12.41

**Table 2:** Phenolic compounds identified by HPLC–MS in DCM fractions.

Compound Name	*m*/*z*	Ionization Mode	Aerial Parts (mg/L)	Root (mg/L)	LOD/LOQ (mg/L)	U % (k = 2)
Chlorogenic acid	353.0878	Negative	2.964	9.914	0.02/0.06	11.14
Fumaric acid	115.0037	Negative	6.67	0.43	0.05/0.17	11.14
Verbascoside	623.1981	Negative	0.055	0.932	0.03/0.1	12.08
Chicoric acid	473.0726	Negative	0.804	4.201	0.03/0.1	10.97
Caffeic acid	179.0350	Negative	16.838	20.563	0.08/0.27	11.07
Vanillic acid	167.0350	Negative	17.34	208.73	0.1/0.33	11.61
Salicylic acid	137.0244	Negative	9.182	13.426	0.01/0.03	11.40
Luteolin	285.0405	Negative	278.48	16.87	0.01/0.03	12.41
Apigenin	269.0456	Negative	53.71	1.825	0.01/0.03	11.54
Hispidulin	301.0707	Positive	7.262	0.689	0.01/0.03	11.23
Chrysin	253.0506	Negative	0.142	0.197	0.01/0.03	11.09

**Table 3:** Compounds obtained from aerial parts PE fraction.

Retention Time (RT)	Compound	Content (%)	RI
23.17	Palmitic acid	1.74	1968
25.86	Linoleic acid	0.77	2156
39.03	Campesterol	0.70	2632
40.12	β-sitosterol	1.21	2731
40.35	Clionasterol	3.33	2731
40.94	α-amirin	5.83	2873
41.02	Lupeol	4.17	2848
41.41	Lanosterol	3.05	2882
41.65	Taraxasterol	21.95	2868
42.94	Betulin	0.92	3090
43.15	α-amirin acetate	12.18	3013
43.34	Lupeol acetate	16.66	2987
	Others	28.26	

**Table 4:** Compounds obtained from root PE fraction.

Retention Time (RT)	Compound	Content (%)	RI
23.17	Palmitic acid	2.16	1968
25.76	Linoleic acid	1.29	2156
39.14	Campesterol	0.76	2632
39.55	Stigmasterol	0.87	2739
40.13	β-sitosterol	1.11	2731
40.35	Clionasterol	2.47	2731
40.69	Germanicol	7.89	2886
40.94	α-amirin	5.12	2873
41.02	Lupeol	4.55	2848
41.41	Lanosterol	3.39	2882
41.65	Taraxasterol	17.10	2868
42.22	Taraxerol	2.97	2886
42.74	Epi-psi- taraxastanonol	1.54	2984
42.94	Betulin	3.02	3090
43.15	α-amirin acetate	14.10	3013
43.35	Lupeol acetate	17.29	2987
	Others	14.37	

**Table 5:** COX-1 and COX-2 inhibitory activities of EA, BuOH, DCM, and PE fractions from aerial parts and roots of *T. mirabile*.

Fractions	COX-1 Inhibition (%)	COX-2 Inhibition (%)
	10 mg/mL	10 mg/mL
Aerial parts EA	76.23 ± 2.60	90.00 ± 2.33
Aerial parts BuOH	79.61 ± 1.13	86.48 ± 0.42
Aerial parts DCM	74.82 ± 0.31	91.06 ± 7.32
Aerial parts PE	80.23 ± 3.24	94.34 ± 0.93
Root EA	80.39 ± 5.18	86.79 ± 5.69
Root BuOH	80.60 ± 5.18	89.98 ± 2.30
Root DCM	81.40 ± 2.19	94.72 ± 1.71
Root PE	86.95 ± 1.13	96.72 ± 0.91
**Standard**	**5 μg/mL**	**50 μg/mL**
Indomethacin	86.02 ± 0.71	99.74 ± 2.09

The data are presented as mean ± standard deviation (*n* = 3).

**Table 6:** The quantity of phenolic compounds and the antioxidant activities of fractions expressed as EC_50_, TEAC, and FRAP values.

Fractions	Phenolic Compounds (mg GAE/g Extract)	Antioxidant Activity			
		DPPH EC_50_ (mg/mL) ^A^	ABTS EC_50_ (mg/mL) ^A^	* TEAC (mM/L) ^B^	* FRAP (mM/L) ^C^
**Aerial EA**	15.97 ± 1.88 ^c^	1.47 ± 0.16 ^a^	1.77 ± 0.17 ^a^	1.954 ± 0.010 ^a^	3.714 ± 0.18 ^a^
**Aerial BuOH**	45.03 ± 5.08 ^a^	5.74 ± 0.31 ^b^	11.53 ± 0.97 ^b^	1.113 ± 0.032 ^b^	0.968 ± 0.11 ^b^
**Aerial DCM**	49.53 ± 2.02 ^a^	3.71 ± 0.39 ^c^	2.91 ± 0.19 ^c^	1.828 ± 0.069 ^a^	3.399 ± 0.27 ^a^
**Aerial PE**	26.93 ± 1.02 ^b^	12.36 ± 0.50 ^d^	16.72 ± 1.80 ^d^	0.815 ± 0.074 ^c^	0.761 ± 0.05 ^b^
**Root EA**	8.25 ± 1.20 ^f^	2.39 ± 0.24 ^e^	3.26 ± 0.37 ^c^	1.798 ± 0.004 ^d^	3.534 ± 0.02 ^a^
**Root BuOH**	31.29 ± 0.93 ^d^	6.46 ± 0.23 ^f^	4.27 ± 0.22 ^e^	1.713 ± 0.048 ^d^	1.933 ± 0.11 ^c^
**Root DCM**	36.39 ± 3.16 ^a,d^	5.73 ± 0.25 ^b^	2.92 ± 0.26 ^c^	1.725 ± 0.069 ^d^	2.570 ± 0.09 ^d^
**Root PE**	21.13 ± 1.10 ^e^	N.d.	N.d.	N.d.	0.379 ± 0.02 ^e^
**Quercetin**	N.c.	0.065 ± 0.008 ^g^	0.053 ± 0.003 ^f^	2.032 ± 0.020 ^e^	3.778 ± 0.02 ^a^

^A^ EC_50_ values have been expressed as the amount of antioxidant required to show 50% DPPH and ABTS radical cation scavenging activity. ^B^ TEAC values are expressed as mM Trolox equivalents. ^C^ The FRAP value is expressed as mM Fe (II) ion equivalents. * The TEAC and FRAP values of the extracts at a concentration of 10 mg/mL and quercetin at a concentration of 0.31 mg/mL are shown. The data are presented as mean ± standard deviation. The significance threshold has been set at *p* < 0.05. Different lowercase letters within the same column indicate that the data are significantly different. N.c.: Not conducted. N.d.: Not determined.

**Table 7:** HPLC gradient method.

Gradient Time	Flow (mL/min)	B%
0.00	0.35	50
1.00	0.35	50
3.00	0.35	100
6.00	0.35	100
7.00	0.35	50
10.00	0.35	50

## Data Availability

Data are contained within the article and are available on request.

## Supplementary Materials

The following supporting information can be downloaded at: https://www.mdpi.com/article/10.3390/plants13233304/s1, Table S1: ^1^H-NMR spectra of the obtained phenolic compounds; Figure S1: UV-Vis Findings and ^1^H-NMR spectra.

## References

[1] Dias M.I., Barros L., Alves R.C., Oliveira M.B.P.P., Santos-Buelga C., Ferreira I.C.F.R. (2014). Nutritional Composition, Antioxidant Activity and Phenolic Compounds of Wild Taraxacum Sect. Ruderalia. Food Res. Int..

[2] Hu C. (2018). Taraxacum: Phytochemistry and Health Benefits. Chin. Herb. Med..

[3] Sharifi-Rad M., Roberts T.H., Matthews K.R., Bezerra C.F., Morais-Braga M.F.B., Coutinho H.D.M., Sharopov F., Salehi B., Yousaf Z., Sharifi-Rad M. (2018). Ethnobotany of the Genus Taraxacum—Phytochemicals and Antimicrobial Activity. Phytother. Res..

[4] Davis P.H. (1975). Flora of Turkey and the East Aegean Islands Volume 5.

[5] Baytop T. (1999). Türkiye’de Bitkilerle Tedavi.

[6] Schütz K., Carle R., Schieber A. (2006). Taraxacum—A Review on Its Phytochemical and Pharmacological Profile. J. Ethnopharmacol..

[7] Martinez M., Poirrier P., Chamy R., Prüfer D., Schulze-Gronover C., Jorquera L., Ruiz G. (2015). *Taraxacum officinale* and Related Species—An Ethnopharmacological Review and Its Potential as a Commercial Medicinal Plant. J. Ethnopharmacol..

[8] Sarı A., Özsoy N., Karahüseyin S. (2020). Investigation of Antioxidant Activity of the Plant Taraxacum Farinosum Hausskn. & Bornm. J. Adv. Res. Health Sci..

[9] Önal S., Timur S., Okutucu B., Zihnioǧlu F. (2005). Inhibition of α-Glucosidase by Aqueous Extracts of Some Potent Antidiabetic Medicinal Herbs. Prep. Biochem. Biotechnol..

[10] Fan M., Zhang X., Song H., Zhang Y. (2023). Dandelion (Taraxacum Genus): A Review of Chemical Constituents and Pharmacological Effects. Molecules.

[11] Di Napoli A., Zucchetti P. (2021). A Comprehensive Review of the Benefits of *Taraxacum officinale* on Human Health. Bull. Natl. Res. Cent..

[12] Jung Y., Ahn Y.G., Kim H.K., Moon B.C., Lee A.Y., Ryu D.H., Hwang G.S. (2011). Characterization of Dandelion Species Using ^1^H NMR- and GC-MS-Based Metabolite Profiling. Analyst.

[13] Grauso L., Emrick S., Bonanomi G., Lanzotti V. (2019). Metabolomics of the Alimurgic Plants *Taraxacum officinale*, Papaver Rhoeas and *Urtica dioica* by Combined NMR and GC–MS Analysis. Phytochem. Anal..

[14] Huber M., Triebwasser-Freese D., Reichelt M., Heiling S., Paetz C., Chandran J.N., Bartram S., Schneider B., Gershenzon J., Erb M. (2015). Identification, Quantification, Spatiotemporal Distribution and Genetic Variation of Major Latex Secondary Metabolites in the Common Dandelion (*Taraxacum officinale* Agg.). Phytochemistry.

[15] Ivanov I., Petkova N., Tumbarski J., Dincheva I., Badjakov I., Denev P., Pavlov A. (2018). GC-MS Characterization of n-Hexane Soluble Fraction from Dandelion (*Taraxacum officinale* Weber Ex F.H. Wigg.) Aerial Parts and Its Antioxidant and Antimicrobial Properties. Z. Naturforsch. C.

[16] Sasikala P., Ganesan S., Jayaseelan T., Azhagumadhavan S., Padma M., Senthilkumar S., Mani P. (2019). Phytochemicals and GC–MS Analysis of Bioactive Compounds Present in Ethanolic Leaves Extract of *Taraxacum officinale* (L). J. Drug Deliv. Ther..

[17] Shi S., Zhao Y., Zhou H., Zhang Y., Jiang X., Huang K. (2008). Identification of Antioxidants from Taraxacum Mongolicum by High-Performance Liquid Chromatography-Diode Array Detection-Radical-Scavenging Detection-Electrospray Ionization Mass Spectrometry and Nuclear Magnetic Resonance Experiments. J. Chromatogr. A.

[18] Shi S.Y., Zhang Y.P., Zhou H.H., Huang K.L., Jiang X.Y. (2010). Screening and Identification of Radical Scavengers from Neo-Taraxacum Siphonanthum by Online Rapid Screening Method and Nuclear Magnetic Resonance Experiments. J. Immunoass. Immunochem..

[19] Ivanov I.G. (2014). Polyphenols Content and Antioxidant Activities of *Taraxacum officinale* F.H. Wigg (Dandelion) Leaves. Int. J. Pharmacogn. Phytochem. Res..

[20] Kassim Ghaima K., Makie Hashim N., Abdalrasool Ali S. (2013). Antibacterial and Antioxidant Activities of Ethyl Acetate Extract of Nettle (*Urtica dioica*) and Dandelion (*Taraxacum officinale*). J. Appl. Pharm. Sci..

[21] Laranjeira C., Nogueira A., Almeida R., Oliveira A., Ferraz Oliveira R., Pinho C., Cruz A. (2017). Antioxidant Activity and Cytotoxicity of Taraxacum Hispanicum Aqueous and Ethanolic Extracts on HepG2 Cells. Int. J. Pharmacogn. Phytochem. Res..

[22] Miłek M., Marcinčáková D., Legáth J., Miłek M., Marcinčáková D., Legáth J. (2019). Polyphenols Content, Antioxidant Activity, and Cytotoxicity Assessment of *Taraxacum officinale* Extracts Prepared through the Micelle-Mediated Extraction Method. Molecules.

[23] Duan L., Zhang C., Zhao Y., Chang Y., Guo L. (2020). Comparison of Bioactive Phenolic Compounds and Antioxidant Activities of Different Parts of Taraxacum Mongolicum. Molecules.

[24] Ionescu D., Predan G., Rizea G.D., Mihele D., Dune A., Ivopol G., Ioniţa C. (2013). Antimicrobial Activity of Some Hydroalcoholic Extracts of Artichoke (*Cynara scolymus*), Burdock (*Arctium lappa*) and Dandelion (*Taraxacum officinale*). Bull. Transilv. Univ. Bras. Ser. II For. Wood Ind. Agric. Food Eng..

[25] (2002). Reference Method for Broth Dilution Antifungal Susceptibility Testing of Yeasts, Approved Standard—Second.

[26] (2006). MA Methods for Dilution Antimicrobial Susceptibility Tests for Bacteria That Grow Aerobically.

[27] Brand-Williams W., Cuvelier M., Berset C., Benzie I., Strain J. (1995). Use of a Free Radical Method to Evaluate Antioxidant Activity. LWT.

[28] Re R., Pellegrini N., Proteggente A., Pannala A., Yang M., Rice-Evans C. (1999). Antioxidant Activity Applying an Improved ABTS Radical Cation Decolorization Assay. Free Radic. Biol. Med..

[29] Benzie I.F.F., Strain J.J. (1996). The Ferric Reducing Ability of Plasma (FRAP) as a Measure of “Antioxidant Power”: The FRAP Assay. Anal. Biochem..

[30] Slinkard K., Singleton V. (1977). Total Phenol Analyses: Automation and Comparison with Manual Methods. Am. J. Enol. Vitic..

