# Supplementary material for: Investigations on the Endemic Species Taraxacum mirabile Wagenitz: HPLC–MS and GC–MS Studies, Evaluation of Antioxidant, Anti-Inflammatory, and Antimicrobial Properties, and Isolation of Several Phenolic Compounds
Source: Plants (Basel). 2024 Nov 25;13(23):3304. doi: 10.3390/plants13233304 (PMC11644681; doi:10.3390/plants13233304)
Supplement: Supplementary file 1 [file plants-13-03304-s001.zip › Figure S1. UV-Vis Findings and H-NMR spectra.pdf]

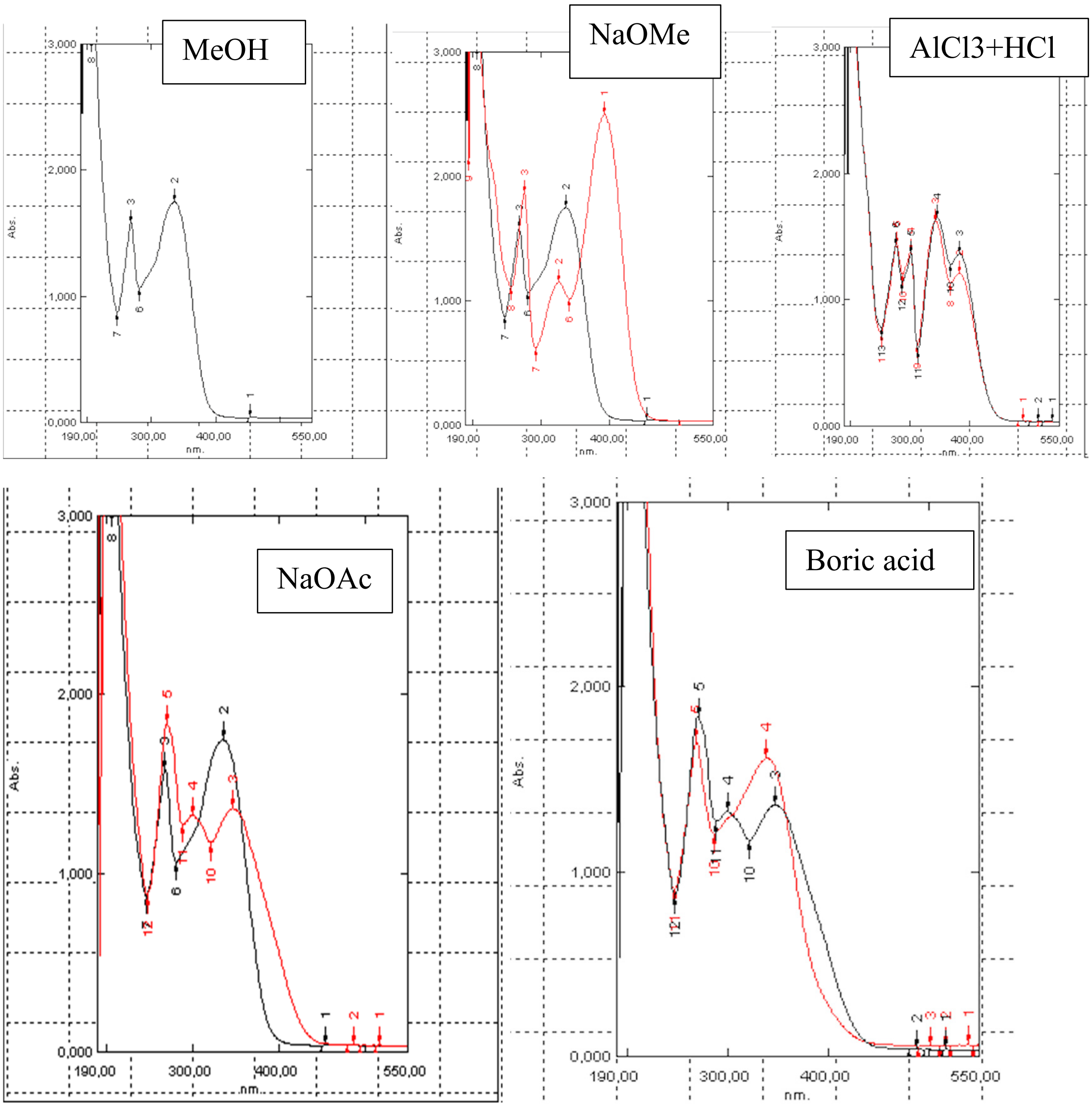

	Band 1 (λ_{max}, nm)	Band 2 (λ_{max}, nm)	Comment
MeOH	336	268	Flavon
MeOH+NaOMe	392	276	4'-OH,7-OH
MeOH+$AlCl_3$	383	277	No 3', 4'-OH

MeOH+ $AlCl_3$+HCl	382	278	5-OH
MeOH+NaOAc	347	271	7-OH
MeOH+NaOAc+H_3BO_3	338	269	No o- dihydroxy

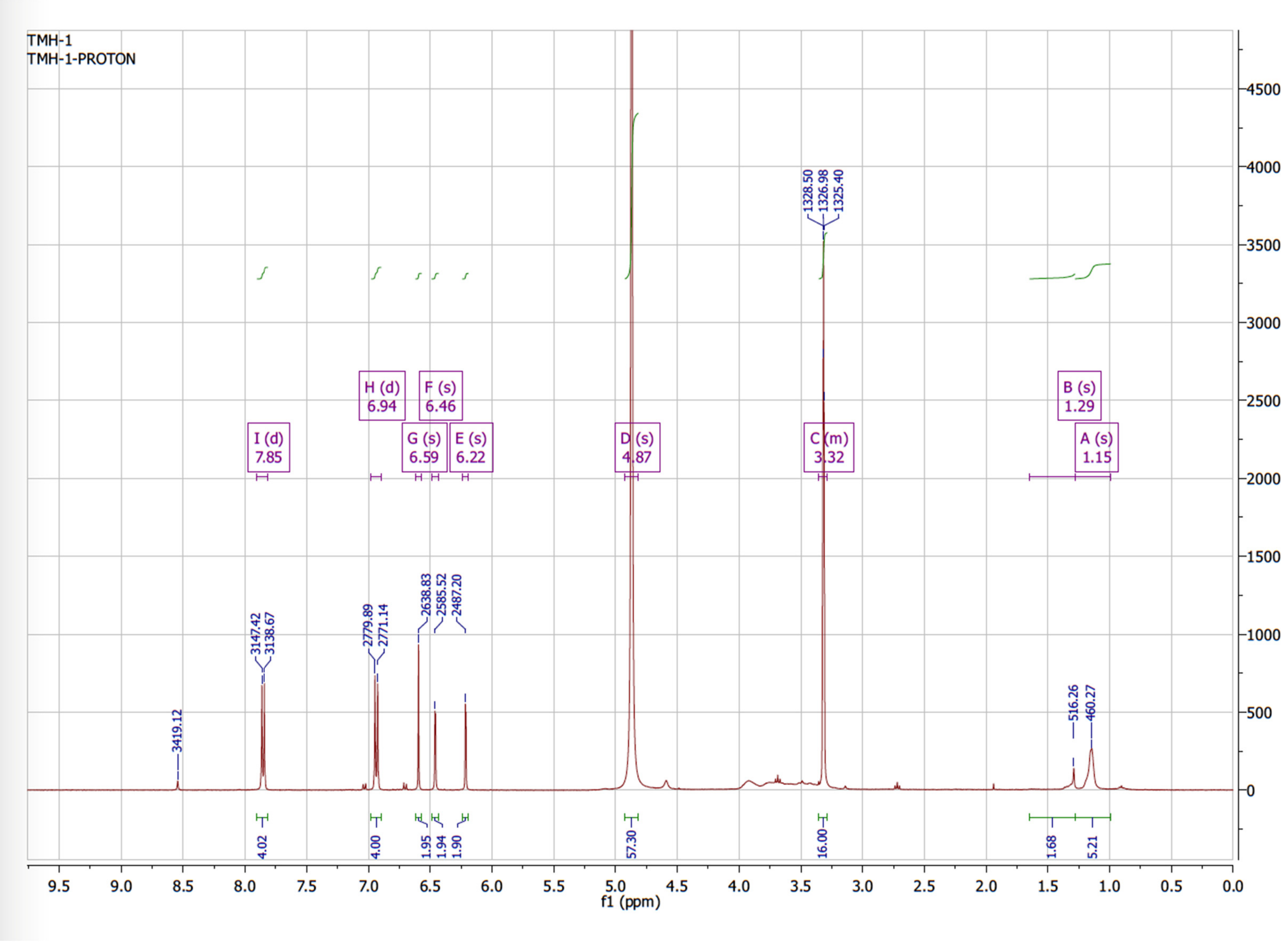
TMH-1
TMH-1-PROTON
I (d)
7.85
H (d)
6.94
G (s)
6.59
F (s)
6.46
E (s)
6.22
D (s)
4.87
C (m)
3.32
B (s)
1.29
A (s)
1.15
3419.12
3147.42
3138.67
2779.89
2771.14
2638.83
2585.52
2487.20
1328.50
1326.98
1325.40
516.26
460.27
4.02
4.00
1.95
1.94
1.90
57.30
16.00
1.68
5.21
f1 (ppm)

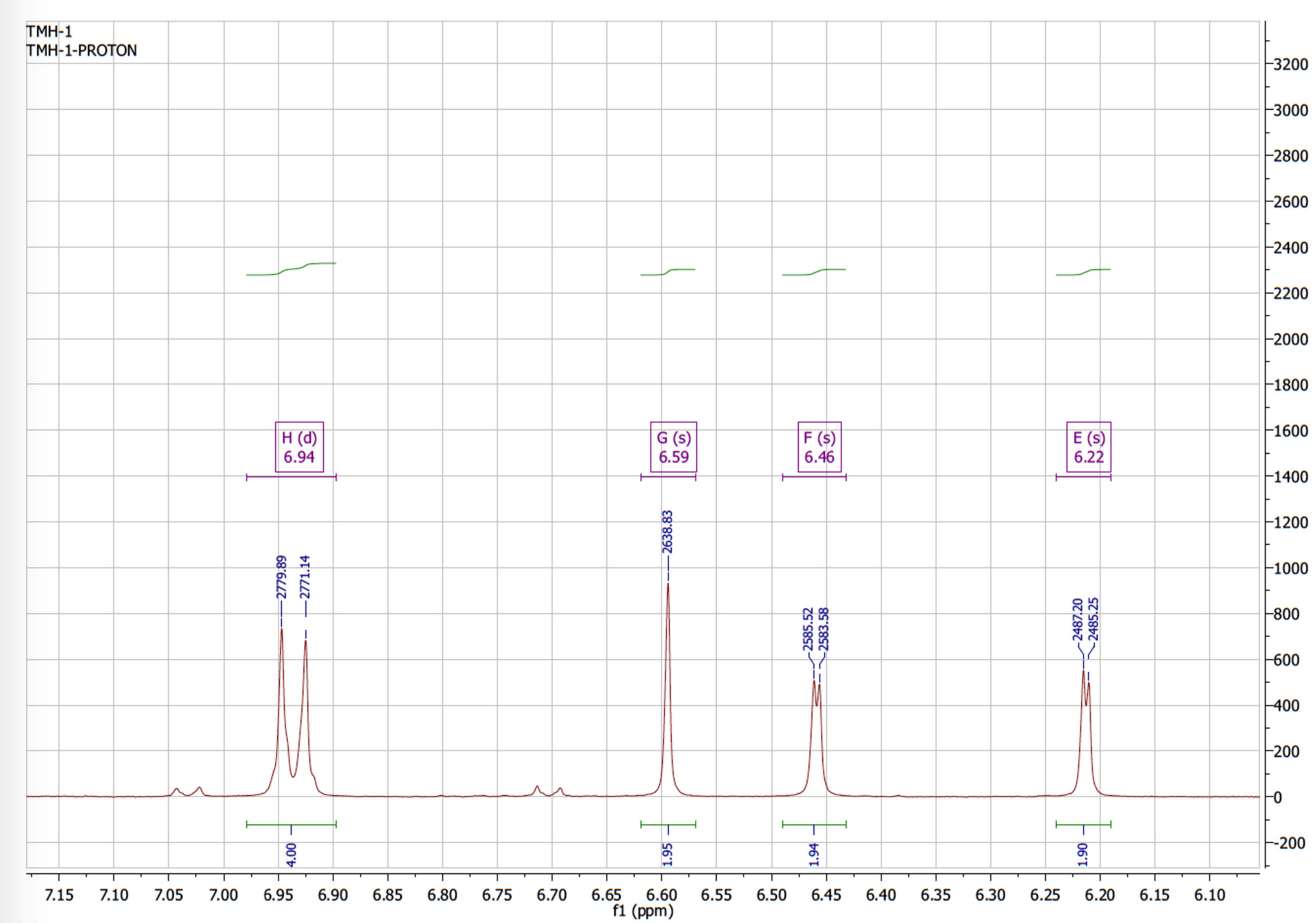

TMH-1
TMH-1-PROTON
H (d)
6.94
G (s)
6.59
F (s)
6.46
E (s)
6.22
2779.89
2771.14
2638.83
2585.52
2583.58
2487.20
2485.25
4.00
1.95
1.94
1.90
f1 (ppm)

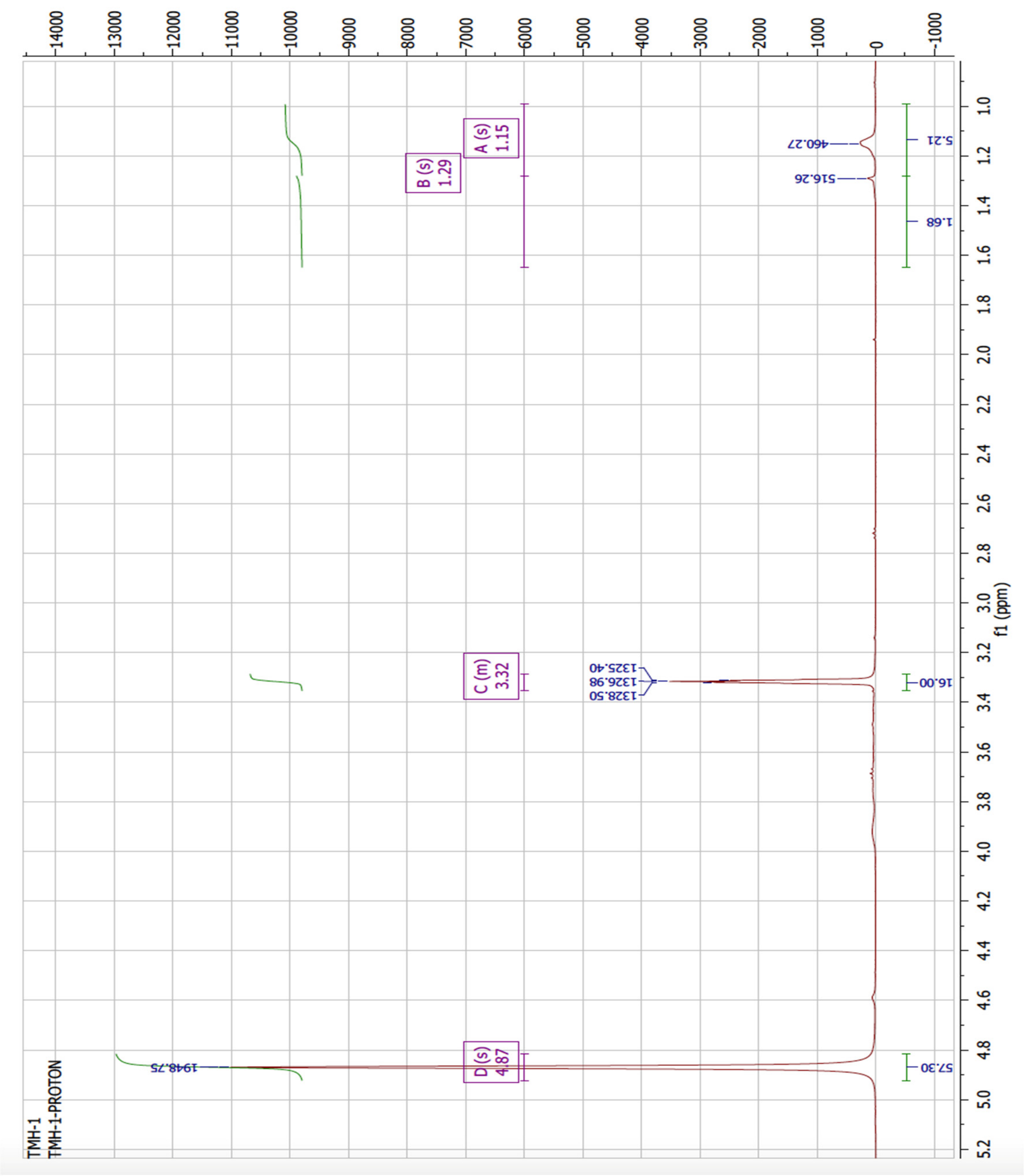
TMH-1
TMH-1-PROTON
A (s) 1.15
B (s) 1.29
C (m) 3.32
D (s) 4.87
460.27
516.26
1325.40
1326.98
1328.50
1948.75
5.21
1.68
16.00
57.30
f1 (ppm)

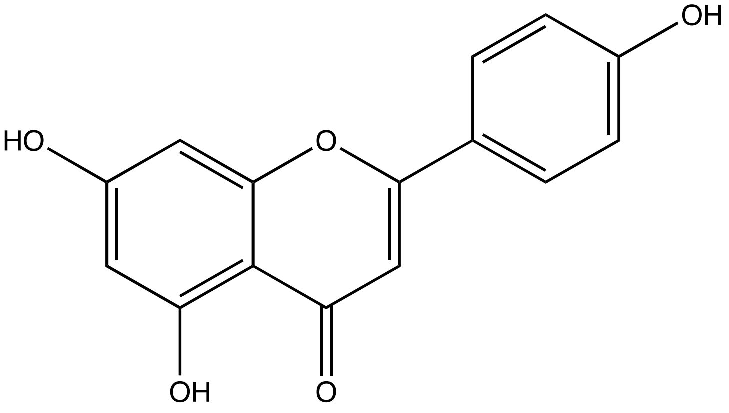
HO
O
OH
OH
O

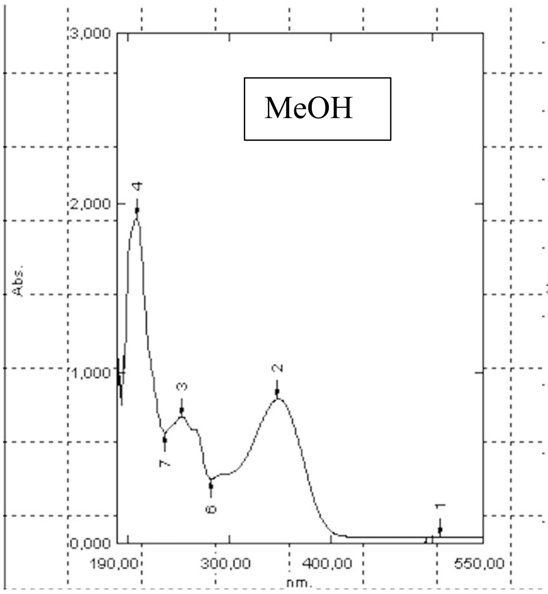
MeOH
3,000
2,000
1,000
0,000
Abs.
190,00
300,00
400,00
550,00
nm.

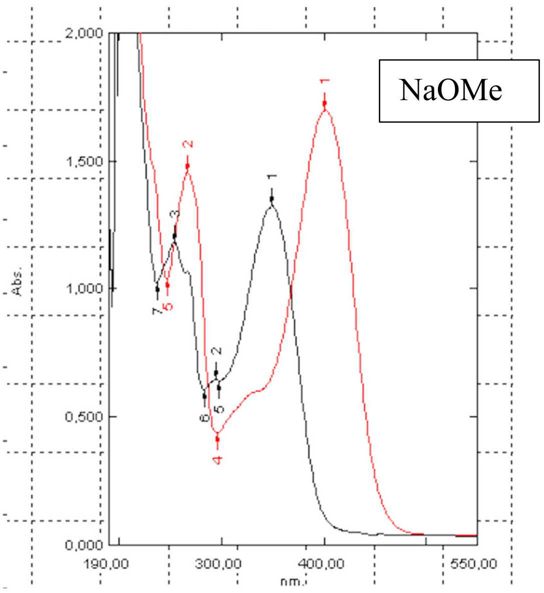
NaOMe
2,000
1,500
1,000
0,500
0,000
Abs.
190,00
300,00
400,00
550,00
nm.

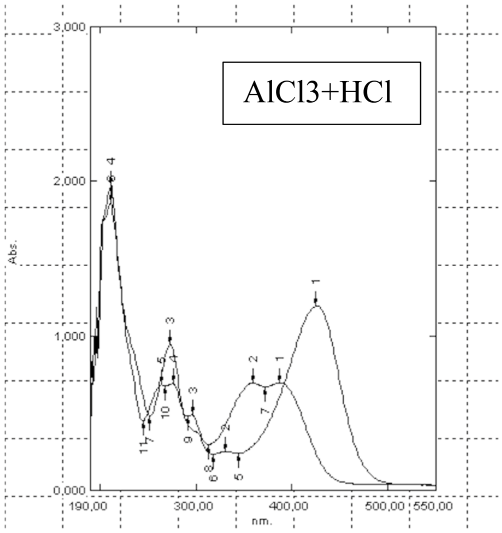
AlCl3+HCl
3,000
2,000
1,000
0,000
Abs.
190,00
300,00
400,00
500,00
550,00
nm.

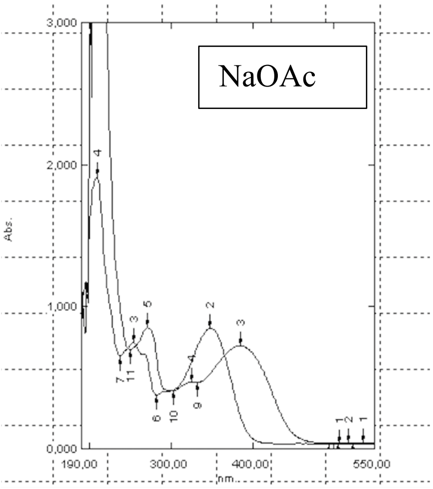
NaOAc
3,000
2,000
1,000
0,000
Abs.
190,00
300,00
400,00
550,00
nm.

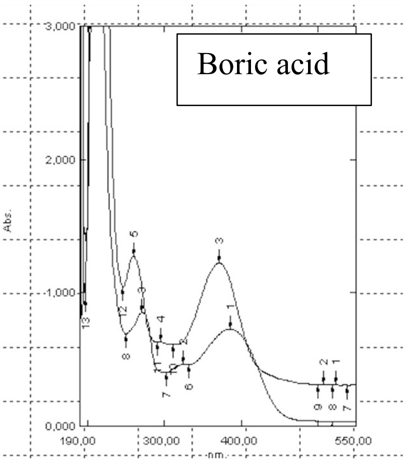
Boric acid
3,000
2,000
1,000
0,000
Abs.
190,00
300,00
400,00
550,00
nm.

	**Band 1 (λ_{max}, nm)**	**Band 2 (λ_{max}, nm)**	**Comment**
MeOH	349	254	Flavon
MeOH+NaOMe	400	266	4'-OH,7-OH
MeOH+$AlCl_3$	426	273	o-dihydroxy
MeOH+ $AlCl_3$+HCl	386	276	5-OH
MeOH+NaOAc	353	269	7-OH
MeOH+NaOAc+H_3BO_3	364	259	o-dihydroxy

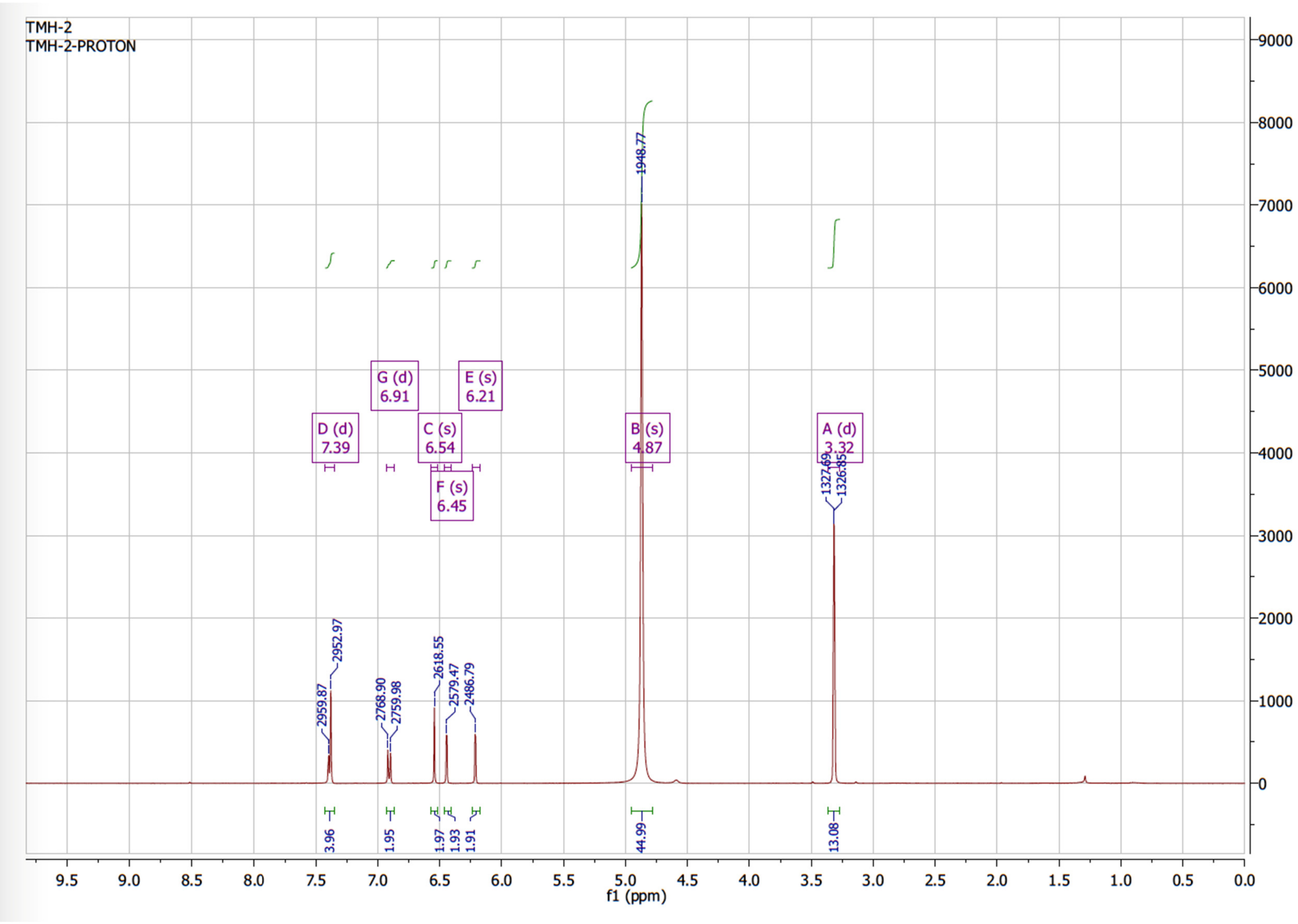
TMH-2
TMH-2-PROTON
D (d) 7.39
G (d) 6.91
C (s) 6.54
F (s) 6.45
E (s) 6.21
B (s) 4.87
A (d) 3.32
2959.87
2952.97
2768.90
2759.98
2618.55
2579.47
2486.79
1948.77
1327.69
1326.85
3.96
1.95
1.97
1.93
1.91
44.99
13.08
f1 (ppm)

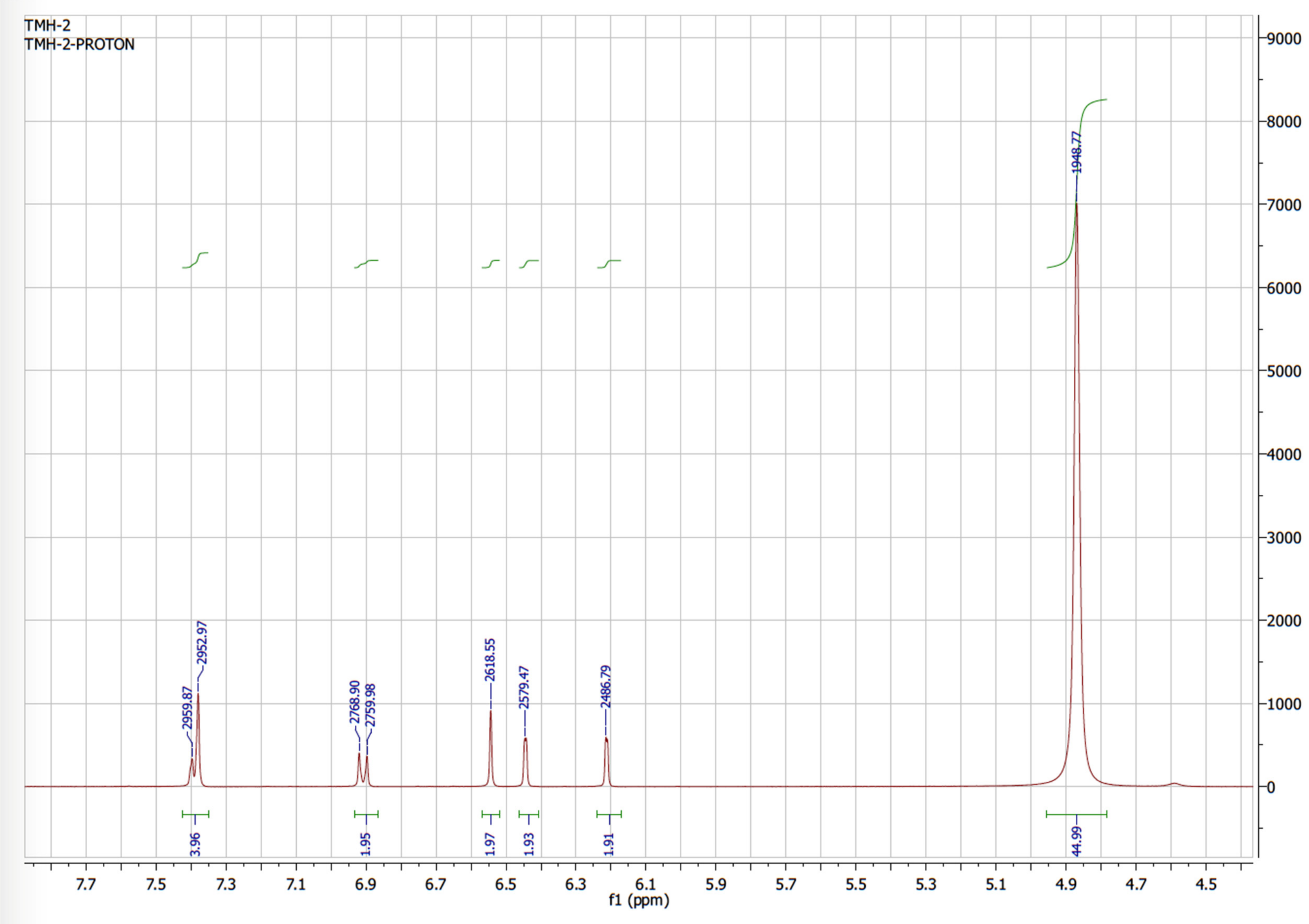

TMH-2
TMH-2-PROTON
2959.87
2952.97
2768.90
2759.98
2618.55
2579.47
2486.79
1948.77
3.96
1.95
1.97
1.93
1.91
44.99
7.7
7.5
7.3
7.1
6.9
6.7
6.5
6.3
6.1
5.9
5.7
5.5
5.3
5.1
4.9
4.7
4.5
f1 (ppm)
9000
8000
7000
6000
5000
4000
3000
2000
1000
0

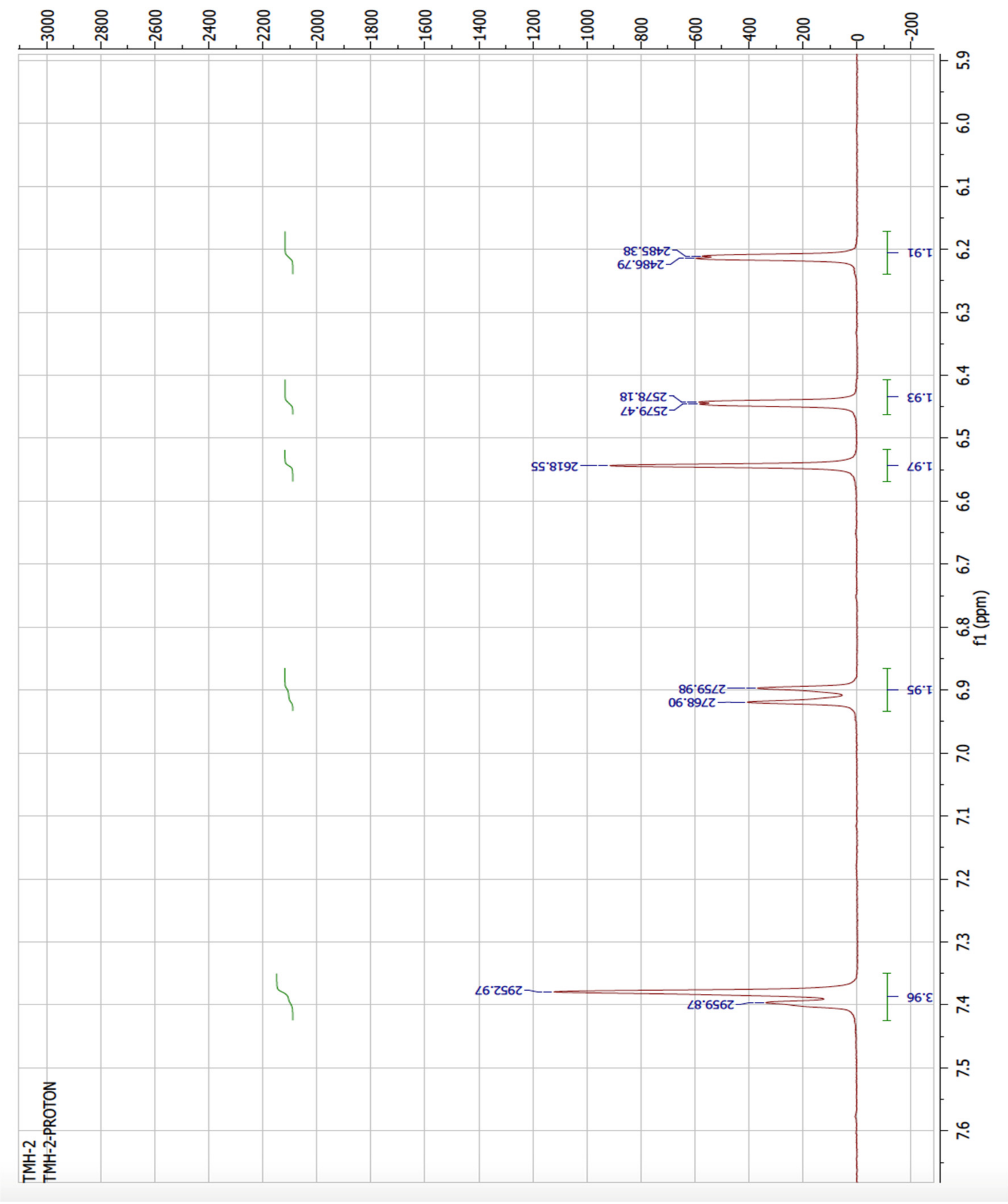
TMH-2
TMH-2-PROTON
f1 (ppm)
2485.38
2486.79
2578.18
2579.47
2618.55
2759.98
2768.90
2952.97
2959.87
1.91
1.93
1.97
1.95
3.96

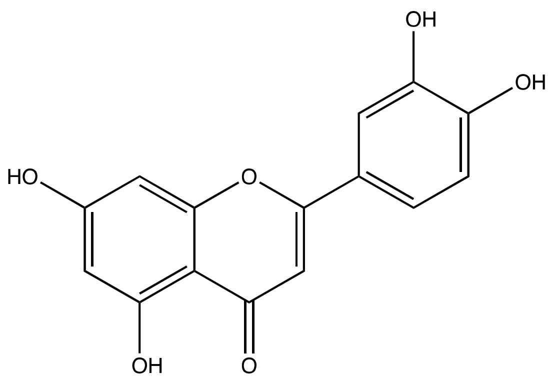
OH
OH
HO
O
OH
O

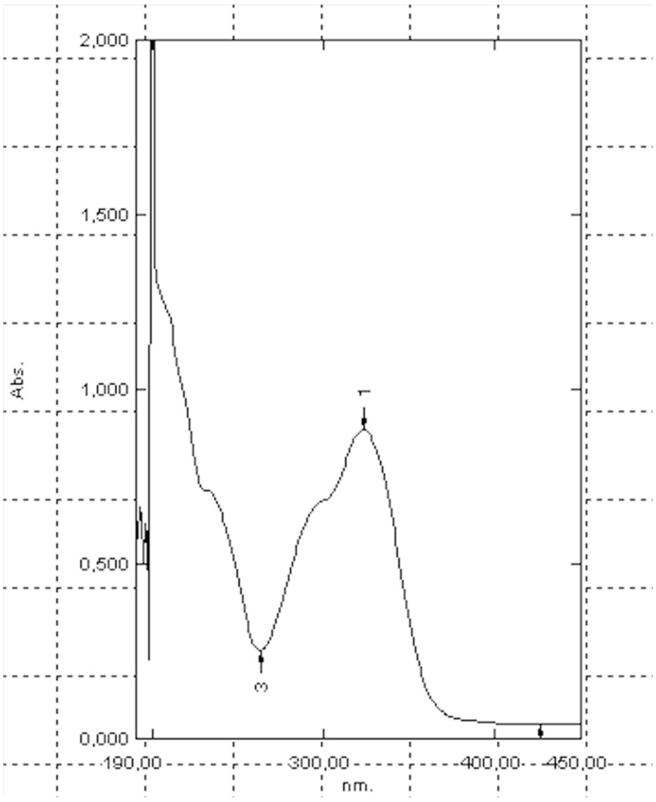

Number	λ_{max}, nm
1	325
2	297
3	234
4	216

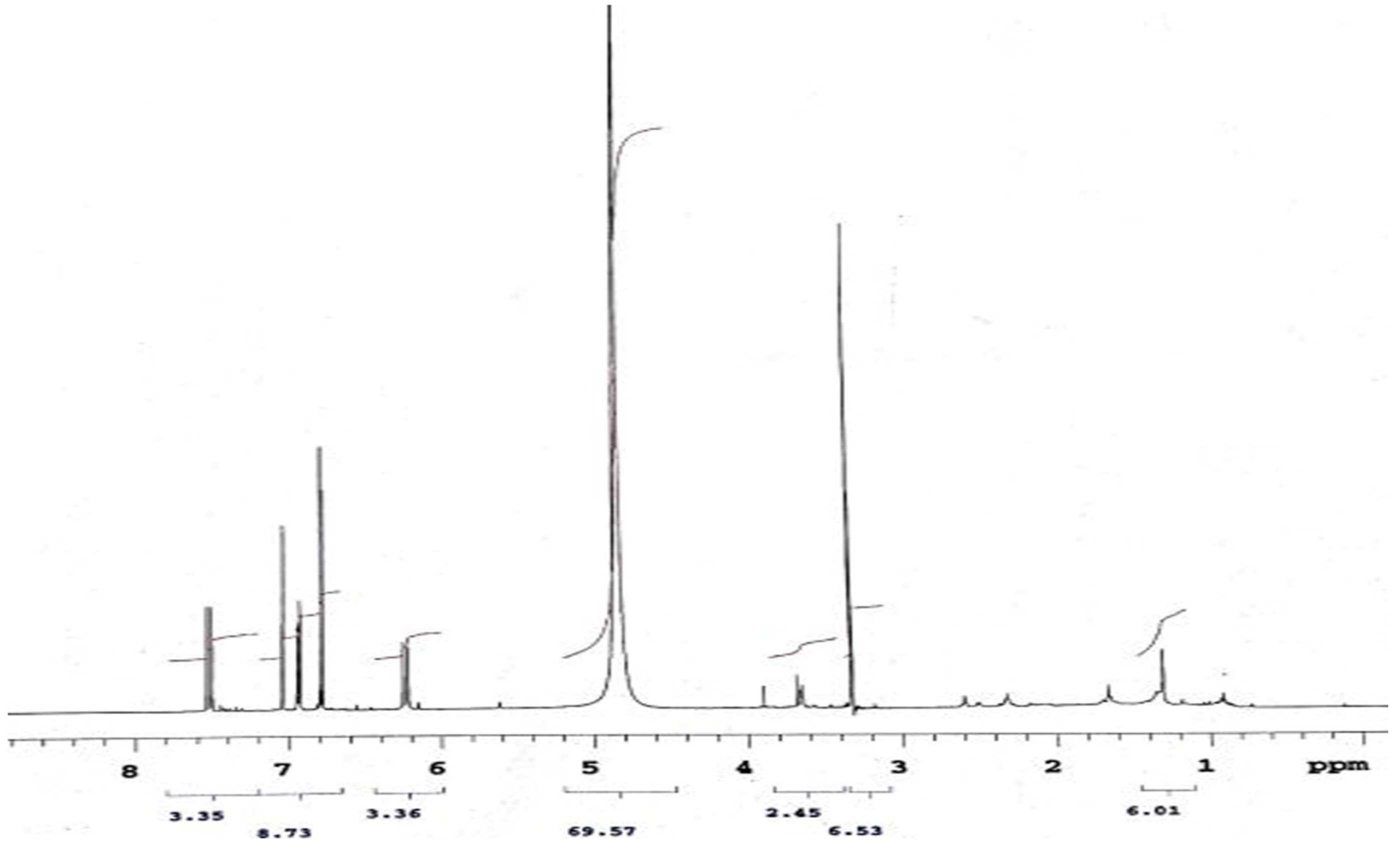
8
7
6
5
4
3
2
1
ppm
3.35
8.73
3.36
69.57
2.45
6.53
6.01

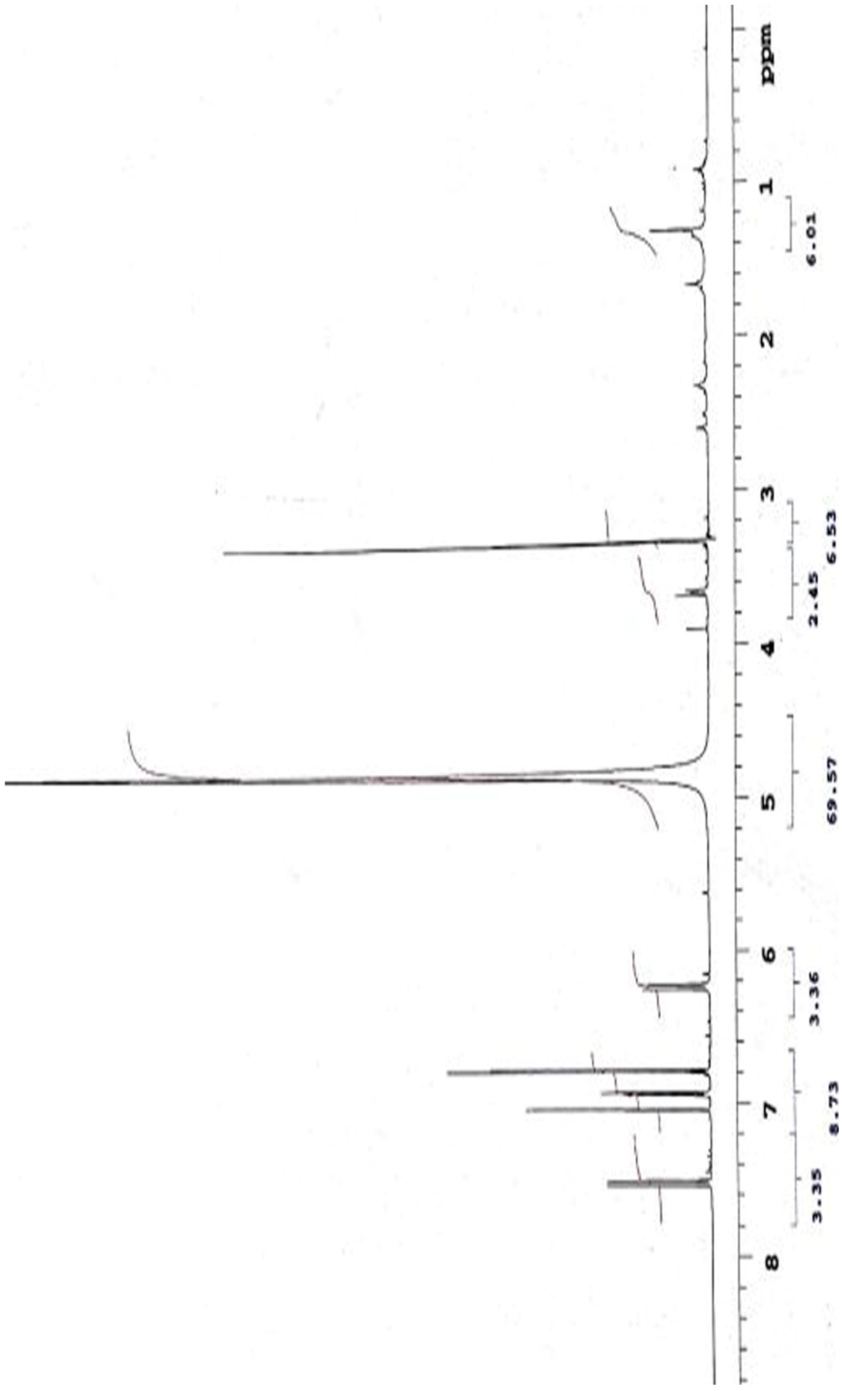
ppm
1
2
3
4
5
6
7
8
6.01
6.53
2.45
69.57
3.36
8.73
3.35

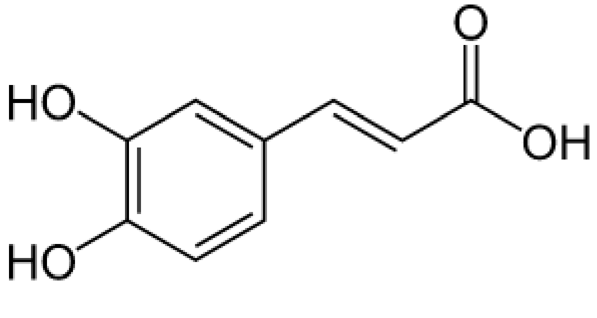
HO
HO
O
OH
