# Supplementary material for: Investigations on the Endemic Species Taraxacum mirabile Wagenitz: HPLC–MS and GC–MS Studies, Evaluation of Antioxidant, Anti-Inflammatory, and Antimicrobial Properties, and Isolation of Several Phenolic Compounds
Source: Plants (Basel). 2024 Nov 25;13(23):3304. doi: 10.3390/plants13233304 (PMC11644681; doi:10.3390/plants13233304)
Supplement: Supplementary file 1 [file plants-13-03304-s001.zip › Table S1. 1H-NMR spectra of the obtained phenolic compounds..pdf]

Table S1. ^{1}H-NMR spectra of the obtained phenolic compounds.

Code	Position	δ_H (J Value, Hz)	Compound Name
TMH-1	1	-	Apigenin
	2	-	
	3	6.59 s	
	4	-	
	5	-	
	6	6.22 d (1.95)	
	7	-	
	8	6.46 d (1.95)	
	1′	-	
	2′ and 6′	7.85 d (8.75)	
	3′ and 5′	6.94 d (8.75)	
	4′	-	
TMH-2	2	-	Luteolin
	3	6.54 s	
	4	-	
	5	-	
	6	6.21 d (1.41)	
	7	-	
	8	6.45 d (1.29)	
	2′	7.38 d (2)	
	3′	-	
	4′	-	
	5′	6.9 d (8.92)	
	6′	7.39 dd (2; 8)	
TMH-17	1	-	Caffeic acid
	2	7.04 d (1.96)	
	3	-	
	4	-	
	5	6.78 d (8.3)	
	6	6.94 dd (8.3; 1.96)	
	7	7.52 d (16.1)	
	8	6.23 d (16.1)	
	COOH	-	

* d= doublet; dd= double doublet; m= multiplet; s= singlet
